# Longer postpartum hospitalization options – who stays, who leaves, what changes?

**DOI:** 10.1186/1471-2393-5-13

**Published:** 2005-10-14

**Authors:** Susan Watt, Wendy Sword, Paul Krueger

**Affiliations:** 1 School of Social Work, McMaster University, Hamilton, Ontario, Canada; 2 School of Nursing, McMaster University, Hamilton, Ontario, Canada; 3 Department of Clinical Epidemiology & Biostatistics, McMaster University, Hamilton and Senior Research Associate, St. Joseph's Health System Research Network, Brantford, Ontario, Canada

## Abstract

**Background:**

This paper examines the practice implications of a policy initiative, namely, offering women in Ontario Canada up to a 60-hour postpartum in-hospital stay following an uncomplicated vaginal delivery. This change was initiated out of concern for the effects of 'early' discharge on the health of mothers and their infants. We examined who was offered and who accepted extended stays, to determine what factors were associated with the offer and acceptance of this option, and the impact that these decisions had on post-discharge health status and service utilization of mothers and infants.

**Methods:**

The data reported here came from two related studies of health outcomes and service utilization of mothers and infants. Data were collected from newly delivered mothers who had uncomplicated vaginal deliveries. Questionnaires prior to discharge and structured telephone interviews at 4-weeks post discharge were used to collect data before and after policy implementation. Qualitative data were collected using focus groups with hospital and community-based health care managers and providers at each site. For both studies, samples were drawn from the same five purposefully selected hospitals. Further analysis compared postpartum health outcomes and post discharge service utilization of women and infants before and after the practice change.

**Results:**

Average length of stay (LOS) increased marginally. There was a significant reduction in stays of <24 hours. The offer of up to a 60-hour LOS was dependent upon the hospital site, having a family physician, and maternal ethnicity. Acceptance of a 60-hour LOS was more likely if the baby had a post-delivery medical problem, it was the woman's first live birth, the mother identified two or more unmet learning needs in hospital, or the mother was unsure about her own readiness for discharge. Mother and infant health status in the first 4 weeks after discharge were unchanged following introduction of the extended stay option. Infant service use also was unchanged but rate of maternal readmission to hospital increased and mothers' use of community physicians and emergency rooms decreased.

**Conclusion:**

This research demonstrates that this policy change was selectively implemented depending upon both institutional and maternal factors. LOS marginally increased overall with a significant decrease in <24-hour stays. Neither health outcomes nor service utilization changed for infants. Women's health outcomes remained unchanged but service utilization patterns changed.

## Background

In this paper, we examine changes in postpartum length of stay (LOS) in five Ontario hospitals following a provincial policy initiative that intended that women be offered the option of up to a 60-hour postpartum stay in hospital after an uncomplicated vaginal birth. In 1999, the Ontario Ministry of Health and Long-Term Care made the decision that, as part of the Healthy Babies Healthy Children program, hospitals should provide women with the option of up to 60 hours of in-hospital postpartum care [1]. The change was initiated out of concern for the impact of 'early' discharge on mother and infant health. The examination of postpartum LOS is based on two research studies, The Ontario Mother and Infant Survey (1998–2000) and The Ontario Mother and Infant Survey II (2001–2004).

In exploring the implementation of this initiative, we examined who was offered and who accepted a 60-hour stay. We compared groups of women who were offered/not offered and accepted/did not accept extended stays to determine what factors were associated with the offer and acceptance of this option. Additionally, we examined the impact of this change on the post-discharge health outcomes and service utilization of mothers and infants.

### Postpartum hospital length of stays

In the 20^th ^century, there was a shift to delivering the majority of infants in hospitals [2,3]. For example, in the United States, the most frequent reason for hospitalization of females is pregnancy [4]. In Canada, the second most prevalent reason for hospitalization of females is pregnancy and childbirth (24% of all women hospitalised) [5]. However, while hospital delivery has become the norm, a search of the literature revealed that the length of the subsequent postpartum hospitalization has been the subject of opinion, conjecture and change.

One constant of postpartum in-hospital LOS over the last century has been the extension and contraction of the time considered appropriate. Variously, LOS has ranged from 14-day lying-in periods [3] to 'drive-through' deliveries [6-8] with only several hours of postpartum in-hospital care.

Over the past 20 years, international literature about postpartum LOS reveals vast practice differences among stakeholders. For example, in reviewing the postpartum discharge practices of eight international clinical trials, Brown, Small, Faber, Krastev, and Davis identified that there was a wide range of definitions of what constituted an 'early discharge' [9]. In 2002, Britton, Baker, Spino and Bernstein reported that, in the United States, 59% of paediatricians surveyed believed that for healthy newborns, a 37 to 48 hour post-delivery hospital stay was optimal and that LOS was a clinical decision that should be determined by the infant's physician [10].

### Changing health policies

The policies of insurers rather than clinicians largely have driven postpartum LOS practices. For example, in the United States in the 1990's, private insurers refused to pay for postpartum hospital stays beyond 12–24 hours, forcing physicians and hospitals to discharge mothers and infants as quickly as possible [2]. Similarly, Brown et al reported that, in Australia, postpartum LOS has decreased significantly in the last 12 years [9]. However, "while the proportion of women with private health coverage who stay in hospital for ≥5 days has fallen, there continues to be only a small number of women in this category who leave hospital before day three (<2% in 1999)" [[9], p. 5].

The literature also describes what happens when institutions attempt to structurally standardize postpartum LOS. As Declercq and Simms make clear, in the United States, the legislative imposition of a minimum 48 hour LOS guarantee was meant to contravene the highly criticized, fiscally driven actions of insurers and the 'drive through' postpartum practices of many medical centres [6].

In the United States, Lichtenstein, Brumfield, Cliver, Chapman, Lenze and Davis observed that "the rules of early discharge, while framed by state and federal laws, were bureaucratically organized to meet hospital priorities" [11]. Lichtenstein et al also explored who was offered and who accepted the offer of an extended stay. They argued that, "early discharge was normative" [[11], p 89] and determined that sociodemographic factors, (i.e., age, education, marital status, urban or rural homes) combined with institutional norms, "produce differential outcomes in the length of postpartum care" [[11], p 89].

On the other hand, single events rather than systematic change, can trigger changes in LOS. In Ontario, the death of an infant after 'early' release, combined with pronouncements by the Canadian Paediatric Society, spurred a policy guarantee of a 60-hour LOS [12,13].

### Changing health practices

The literature about postpartum LOS also revealed differences of opinion among stakeholders regarding the outcomes of 'early' discharge. Usually, the debate has focussed on health of the infant. U.S. paediatricians who took part in the study by Britton et al expressed concern that, because of shortened postpartum LOS, "they had cared for infants who were discharged early and experienced adverse outcomes related to the short stay" [[10], p 53]. D'Amour, Goulet, Labadie, Bernier, and Pineault reported that nurses surveyed in Canada were concerned about release in under 72 hours because "It is often at the third day following birth, when the mother and the baby have returned home, that the main health problems arise" [[14], p 398] and that "shorter stays in hospital often result in parents not having enough time to receive or fully integrate hospital information about care for both the baby and the mother" [[14], p 398]. On the other hand, many articles reported no adverse outcomes because of early postpartum release [9,15,16] and that the relationship between a shorter stay and newborn infant adverse health outcomes, including readmission, are inconsistent [19-27].

## Methods

Data collection for The Ontario Mother and Infant Survey (TOMIS) occurred between November 1998 and June 1999 and was staggered across sites. The Hospital Stay and Postpartum Home Visiting Program extension to the Health Babies Healthy Children program was added in November 1999. TOMIS II data collection was started in September 2001, with initiation staggered across sites, and was completed in March 2002.

Data for TOMIS I and TOMIS II were collected using quantitative cross-sectional surveys completed at discharge (self administered questionnaire) and approximately 4-weeks post-discharge (structured telephone interview). The survey methods and instruments used for TOMIS II paralleled those used in TOMIS, which allowed for an appropriate comparison of data at two points in time. The same study sites, sample size, eligibility criteria, recruitment strategy, and instruments were used for the two surveys [16]. Five purposefully selected Ontario hospitals provided respondents who constituted a cross-section of mothers and newborn infants with diverse socio-economic characteristics and access to varying health and social services. The characteristics of the hospitals were as follows:

Site 1 Southern, suburban, teaching hospital, metropolitan catchment area, 3900 annual births

Site 2 Central east regional centre, urban & rural catchment areas, 1500 annual births

Site 3 Central south regional centre, urban & rural catchment areas, 4500 annual births

Site 4 Southern, urban, teaching, metropolitan catchment area, 2700 annual births

Site 5 Central north regional centre, urban & rural catchment areas, 2000 annual births.

Participants for both studies included the first 250 eligible, consenting women from each site, totalling 1,250 participants in each study. This sample size was determined to be large enough to allow for the examination of many variables together, and was in keeping with the generally accepted guideline of 30 subjects per variable [28]. Women were eligible if they (a) had given birth vaginally to a single live infant, (b) were being discharged from hospital at the same time as their infant, (c) were assuming care of their infant at the time of discharge, and (d) were competent to give consent to participate. Women were excluded if they (a) had an infant who required admission to a neonatal intensive care or special care nursery for more than 24 hours or (b) were unable to communicate in one of the study languages – English, French, Chinese, and Spanish. In previous studies it had been determined that this way of recruiting subjects produced samples at each site that reflected the populations known to be served by each hospital thereby permitting generalizability of the results to this healthy group of women and their newborn children [16]. Each study hospital continued to utilize its own postpartum care protocols throughout the recruitment period. Full descriptions of the methodology have been published [16]. The ethics review committees of McMaster University and each of the hospitals involved in the study granted ethical approval.

Descriptive statistics were computed by site for all variables measured, including frequency counts and percentages, or means and standard deviations (as appropriate). T-tests, chi-square tests or, when appropriate, Fisher's exact tests were used to determine differences between sites or differences between TOMIS and TOMIS II data. Chi-square tests were used to identify variables associated with being offered the option of a 60-hour stay in hospital following delivery and variables associated with acceptance of a 60-hour length of stay. The decision about which variables to include in these bivariate analyses were made *a priori *based on the literature, available data, and clinical judgement. Unadjusted odds ratios, corresponding 95% confidence intervals, and p-values are reported for these associations. Only variables that were found to be statistically significant in the bivariate analyses or were judged to be clinically relevant were included in the logistic regression. Multiple logistic regression analysis was used to identify the best predictors of being offered the option of a 60-hour stay as well as to identify the best predictors of acceptance of a 60-hour stay. The final results are reported as adjusted odds ratios (OR) and 95% confidence intervals. The goodness of fit of the logistic regression model was assessed using the rho-squared statistic [29]. A rho-square value between 0.20 and 0.40 suggests a very good fit of the model. A probability level of <0.05 was used to determine statistical significance. SPSS was used for all statistical computations.

## Results

### Study participants

Of the 1250 women recruited for TOMIS II, 890 (61.2% to 82.8% per site) participated in the follow-up telephone interview at 4-weeks post discharge. Table 1 provides a profile of the women and infants who took part in the survey. The profiles of the samples generally reflect the census profiles of women aged 15 to 35 in the tracts from which each hospital normally draws its patient population. In keeping with ethical standards of research practice in hospitals, data on non-consenting women were not collected

**Table 1:** Characteristics of TOMIS II study participants^a^

Characteristic	Site 1 (n = 250)	Site 2 (n = 250)	Site 3 (n = 250)	Site 4 (n = 250)	Site 5 (n = 250)
Maternal age in years (mean ± SD)^b^	31.7 ± 4.9	28.8 ± 5.1	29.3 ± 5.2	29.7 ± 5.7	27.0 ± 5.1
Gestation in weeks (mean ± SD)	39.5 ± 1.4	39.7 ± 1.4	39.7 ± 1.4	39.4 ± 1.7	39.4 ± 1.3
Birth weight in grams (mean ± SD)^b^	3344 ± 452	3525 ± 516	3564 ± 485	3404 ± 682	3517 ± 557
	**%**	**%**	**%**	**%**	**%**
First live birth	43.4	42.0	40.4	46.0	44.0
Martial status ^c^					
Married	88.8	71.3	79.9	78.3	59.3
Common-law/living with partner	6.0	21.9	14.5	12.3	27.8
Never married/separated/widowed/divorced	5.2	6.9	5.6	9.4	12.9
Family income ^c,d^					
<$20,000	12.1	14.7	7.4	28.5	23.8
$20,000 to $39,999	18.2	20.7	13.0	18.4	19.7
$40,000 to $59,999	17.3	29.7	23.4	16.7	18.8
$60,000 to $79,999	16.0	17.2	22.1	13.6	17.0
≥$80,000	36.4	17.7	34.2	22.8	20.6
Born in Canada ^c^	37.6	93.6	81.1	34.1	96.8
Self-reported ethnicity ^c^					
Canadian	26.9	94.3	79.2	37.0	93.6
Other than Canadian	73.1^d^	5.7	20.8	63.0^f^	6.4
Language spoken at home ^c^					
English/French	55.2	99.6	86.0	63.9	99.6
Other than English/French	44.8^e^	0.4	14.0	36.1^f^	0.4
Highest level of education ^c^					
Less than high school	4.5	9.7	11.6	17.1	13.4
High school	9.7	13.3	14.1	20.8	10.2
Some community college/technical school	5.3	14.5	10.4	8.6	13.4
Completed community college/technical school	19.8	33.5	24.1	17.6	29.7
Some university	10.1	5.6	9.6	6.9	5.3
University	50.6	23.4	30.1	29.0	28.0

^a ^originally published in Sword, W., Watt, S., Krueger, P. (2004). Implementation, Uptake, and Impact of a Provincial Postpartum Program, *Canadian Journal of Nursing Research*, 36, 60–82

^b ^ANOVA indicated a statistically significant difference across sites (p < 0.05)

^c ^Chi-square test indicated a statistically significant difference across sites (p < 0.05)

^d ^8.4% of the total sample did not report family income

^e ^26.9% of the total sample at Site 1 "Chinese"; 15.5% "Jewish"; 23.6% spoke Chinese at home

^f ^11.9% of the total sample at Site 4 "South Asian"; no predominant language "Other than English/French"

There were no statistically significant differences in any of these variables between those women who completed the telephone interview and those who did not. This finding suggests that subjects lost to follow-up were similar in terms of sociodemographic characteristics to those who participated in the interview. Statistically significant differences were found between sites for all of the variables except mean length of gestation and first live birth (which are less likely to vary by site given the inclusion criteria), thereby reflecting the diversity in the sample we had intended to achieve with the selection of study sites. When TOMIS II participants were compared to TOMIS participants, there were minimal differences in sociodemographic characteristics reported in Table 1.

### Implementation of an extended lengths of stay

There were wide and statistically significant differences (p < 0.05) in implementation of the 60-hour stay option across sites, with between 11.7% and 81.2% of women reportedly having been offered an extended hospital stay (Site 1 – 11.7%; Site 2 – 41.9%; Site 3 – 81.2%; Site 4 – 39.9%; Site 5 – 52.3%). Of those women who were offered a 60-hour stay, between 21.1% and 39.4% accepted (Site 1 – 21.1%; Site 2 – 39.4%; Site 3 – 30.4%, Site 4 – 31.3%; Site 5 – 21.3%). No statistically significant difference was found across sites for these acceptance rates.

Implementation challenges in offering a 60-hour stay identified by focus group participants included a limited capacity due to recent downsizing and reorganization of catchment areas for obstetrical units. These factors were compounded by a recent increase in the number of deliveries at some of the sites. At sites where physical capacity was an issue, care providers acknowledged that they did not routinely offer an extended stay but rather made clinical judgments in determining an appropriate LOS for each woman admitted to their unit.

Those women who accepted a longer stay had a variety of reasons for doing so, including their own health (31.7%), the health of their baby (39.8%), and breastfeeding difficulties (20.2%). Those women who declined the 60-hour stay offer identified wanting to go home as the major reason for turning it down (39.5%). Additionally, this group identified readiness for discharge (25.0%), dissatisfaction with their hospital accommodation or care (16.0%), and other children at home (10.2%) as reasons for declining a longer hospital stay.

Satisfaction with postpartum LOS increased significantly following the change in policy and the resultant increase in LOS. Satisfaction increased from 74 to 89% (p < 0.01). Although not statistically significant, women who were not satisfied preferred longer stays (77.3% and 69.2%; p = 0.220). While women who preferred a shorter postpartum stay increased from 21% to 28%, it is interesting to note that of those offered a 60-hour stay, 96.1% were satisfied with their postpartum LOS whether or not they made use of the offer. Conversely, 80% of the women who were not offered a 60-hour stay were dissatisfied with their LOS.

### Offer of an extended length of stay

Based on the literature and the findings of TOMIS, a number of variables were examined to determine what factors might be associated with being offered up to a 60-hour postpartum LOS. Five variables were found to be significantly associated – hospital site, having a family physician, self-defined ethnicity of mother as Canadian, English or French language spoken at home, and being born in Canada (Table 2).

**Table 2:** Variables associated with being offered the option of a 60-hour stay in hospital following delivery ^a ^(n = 1230)

	Offered 60 Hr LOS				
				
Variables	Yes (%)	No (%)	P value ^b^	Unadjusted Odds Ratio	95% Confidence Interval
Information Collected From Mother Prior to Discharge From Hospital					
Site (n = 1230):					
1	19 (7.7)	227 (92.3)		1.00	**-**
4	65 (27.2)	174 (72.8)		4.46	(2.58, 7.72)
2	89 (35.9)	159 (64.1)		6.69	(3.92, 11.42)
5	127 (51.0)	122 (49.0)		12.44	(7.32, 21.13)
3	216 (87.1)	32 (12.9)	<0.001	80.64	(44.3, 146.6)
Age of mother (n = 1228)					
20 to 39	468 (41.7)	653 (58.3)		1.00	-
<20 or >39	47 (43.9)	60 (56.1)	0.663	1.09	(0.73, 1.63)
First live birth (n = 1226):					
No	286 (40.9)	413 (59.1)		1.00	-
Yes	228 (43.3)	299 (56.7)	0.409	1.10	(0.88, 1.38)
Has a family physician (n = 1226):					
No	17 (26.6)	47 (73.4)		1.00	-
Yes	497 (42.8)	665 (57.2)	0.011	2.07	(1.17, 3.64)
Baby had medical problems since birth (n = 1229):					
No	442 (41.1)	634 (58.9)		1.00	-
Yes	74 (48.4)	79 (51.6)	0.087	1.34	(0.96, 1.88)
Number of concerns^c ^prior to discharge (n = 1230):					
Two or more	203 (39.3)	314 (60.7)		1.00	-
One or fewer	313 (43.9)	400 (56.1)	0.104	1.21	(0.96, 1.52)
Mother had medical problems since birth (n = 1229):					
No	478 (41.5)	674 (58.5)		1.00	-
Yes	38 (49.4)	39 (50.6)	0.176	1.37	(0.87, 2.18)
Mother has other children (n = 1224):					
Yes	293 (40.8)	426 (59.2)		1.00	-
No	219 (43.4)	286 (56.6)	0.361	1.11	(0.88, 1.40)
Language spoken most often at home (n = 1230):					
Other	55 (23.8)	176 (76.2)		1.00	-
English or French	461 (46.1)	538 (53.9)	<0.001	2.74	(1.98, 3.80)
Ethnic or cultural group (n = 1216):					
Other	95 (23.6)	307 (76.4)		1.00	-
Canadian	419 (51.5)	395 (48.5)	<0.001	3.42	(2.62, 4.48)
Place of birth (n = 1229):					
Other	99 (26.1)	280 (73.9)		1.00	-
Canada	417 (49.1)	433 (50.9)	<0.001	2.72	(2.09, 3.56)
Marital status (n = 1223):					
Partnered	470 (41.8)	655 (58.2)		1.00	-
No Partner	45 (45.9)	53 (54.1)	0.426	1.18	(0.78, 1.79)
Total income before taxes and deductions of all household members (n = 1134):					
Less than $20,000	70 (36.3)	123 (63.7)		1.00	-
$20,000 or more	406 (43.1)	535 (56.9)	0.078	1.33	(0.97, 1.84)
Highest level of education (n = 1221):					
Completed high school or less	125 (41.4)	177 (58.6)		1.00	-
Education beyond high school	387 (42.1)	532 (57.9)	0.826	1.03	(0.79, 1.34)
Mother feels that help and support at home will meet both her and baby's needs (n = 1220):					
Other response	215 (39.7)	326 (60.3)		1.00	-
Definitely yes	299 (44.0)	380 (56.0)	0.131	1.19	(0.95, 1.50)
Mother feels she and baby are ready to be discharged (n = 1227):					
Definitely/Probably no/Don't know	65 (36.1)	115 (63.9)		1.00	-
Definitely/Probably yes	450 (43.0)	597 (57.0)	0.085	1.33	(0.96, 1.85)

^a ^Exact question asked was, "Were you offered the option of a 60-hour stay in hospital after your delivery? Yes/No".

^b ^Chi-square test

^c ^Concerns included: breast-feeding; bottle-feeding; infant care and behaviour; signs of illness in infant; physical changes and care of yourself; sexual changes and intercourse; emotional changes in yourself.

The final logistic regression model found that the first three factors were the most important predictors of being offered a 60-hour postpartum LOS (Table 3). The hospital in which a woman delivered remained the single most important predictor.

**Table 3:** Final logistic regression model of the most important predictors of being offered a 60-hour stay in hospital following delivery (n = 1212^a^)

Predictors	Adjusted Odds Ratio ^b^	95% Confidence Interval
Site		
1	1.00	-
4	7.37	(4.66, 11.68)
2	13.22	(8.29, 21.09)
5	14.84	(9.13, 24.14)
3	33.68	(33.68, 115.3)
Mother has a family physician:		
No	1.00	-
Yes	2.45	(1.28, 4.69)
Ethnic or cultural group:		
Other	1.00	-
Canadian	1.84	(1.26, 2.70)

Final Logistic Regression Model Statistics: Rho-square = 0.25 (a pseudo R^2^, values between 0.2 and 0.4 suggest a very good fit) Hosmer and Lemeshow Goodness-of-fit test = 0.98 (values greater than 0.25 indicate good fit) 74.3% correctly classified		

^a ^Eighteen of the 1230 (1.5%) mothers had missing values for one or more of the variables included in the final model.

^b ^Odds ratios for categorical variables represent comparisons with the referent group (OR = 1.00) after adjustment for all other variables in the model.

### Acceptance of an extended length of stay

The ten variables found to be associated with the acceptance of the offer are the following:

1. first live birth;

2. the baby having medical problems since birth;

3. the mother having two or more concerns prior to discharge;

4. the mother having medical problems since birth;

5. the mother being unsure that help and support at home will meet both her and the baby's needs;

6. the mother being not sure that she and the baby are ready for discharge;

7. the mother having 2 or more unmet learning needs while in hospital;

8. the mother's rating of her own health as only good/fair/poor since having the baby;

9. the mother having a low affective social support score; and,

10. the mother not being able to tell when her baby is sick (See Additional file 1).

Four of these variables emerged as the best predictors of acceptance – unmet learning needs in hospital, infant medical problems, first live birth, and mother's perception of her own discharge readiness (Table 4).

**Table 4:** Final logistic regression model of the most important predictors of accepting an offer for a 60-hour stay in hospital following delivery (n = 379)

Predictors	Adjusted Odds Ratio ^a^	95% Confidence Interval
Number of unmet identified learning needs while in hospital:		
Less than 2	1.00	-
2 or more	1.98	(1.27, 3.10)
Baby had medical problems since birth ^b^:		
No	1.00	-
Yes	2.82	(1.49, 5.34)
First live birth ^b^:		
No	1.00	-
Yes	1.86	(1.20, 2.89)
Mother feels she and baby are ready to be discharged ^b^:		
Definitely yes	1.00	-
Other response	1.84	(1.19, 2.86)

Final Logistic Regression Model Statistics: Rho-square = 0.10 (a pseudo R^2^, values between 0.2 and 0.4 suggest a very good fit) Hosmer and Lemeshow Goodness-of-fit test = 0.36 (values greater than 0.25 indicate good fit) 64.9% correctly classified		

^a ^Odds ratios for categorical variables represent comparisons with the referent group (OR = 1.00) after adjustment for all other variables in the model.

^b ^Information collected from mother prior to discharge from hospital

### Health outcomes and post-discharge service utilization

The question then arose, "If postpartum LOS has increased, what is the impact of this change on the health status of the mother and infant, and on their service utilization following discharge?" The health status of both the mother and her infant, as reported by the mother, was unchanged from TOMIS. In TOMIS 88.4% and in TOMIS II 90.7% of women reported their own health as excellent, very good, or good (p = 0.12). Infant health was reported in both studies as excellent, very good, and good by 98.0% of mothers (p = 0.33).

Similarly, service utilization in terms of use of community physicians and emergency rooms and hospital readmissions for infants was unchanged (Table 5). Women, however, experienced more hospital readmissions but fewer had contacts with community physicians and emergency rooms (Table 5).

**Table 5:** Service utilization of mother and infant

Service Utilization	TOMIS n = 875		TOMIS II n = 890		p-value
		
	No.	%	No.	%	
Mother					
Community physician ^a^	670	76.6	640	71.9	0.03
ER	59	7.1	29	3.3	0.001
Readmission	8	0.9	35	4.0	<0.001
Infant					
Community physician ^b^	826	94.4	857	96.3	0.076
ER	70	8.0	66	7.4	0.71
Readmission ^c^	35	4.0	40	4.5	0.69

^a ^includes family physicians, gynaecologists, and walk-in clinics; excludes exclusive ER or midwife contact

^b ^includes family physicians, paediatricians, and walk-in clinics; excludes exclusive ER or midwife contact

^c ^excludes day admissions for circumcisions

## Discussion

The results demonstrate that implementation of this policy was dependent upon the specific hospital's approach to postpartum care and what the decision makers in each hospital viewed as an appropriate LOS. The attitudes of providers, namely, physicians and nurses, and the perception of administrators about the adequacy of their facilities to accommodate what they believed would be a significant uptake of the offer of an extended stay played a role in determining whether or not the practice was implemented in each of the sites. It was clear that providers did not perceive the 60-hour stay policy as a practice requirement. Yet the policy appears to have increased the sensitivity of providers to the possible problems associated with a short hospital stay. Hence, stays of <24 hours decreased. It would appear that a new norm developed for a 24 to 48 hour postpartum LOS for uncomplicated vaginal deliveries.

Although providers had been quite concerned about short stays, women were, overall, very satisfied with their LOS whether or not they accepted an extended stay. The anticipated increased demand for extended stays did not materialize. Rather, for a variety of reasons, many women appeared to prefer to leave hospital as soon as they and their infants were able to do so and did not suffer negative health consequences in the following 4 weeks.

The two features of the social location of a mother that would most likely lead to being offered a 60-hour postpartum stay, self-identified ethnicity as Canadian and having a family physician, suggests that women who were offered an extended stay were not marginalized and were likely to have social supports. The women who often are deemed by health professionals as being most at risk for poor outcomes with early discharge, the young, the poor, and the unsupported, did not appear to be targeted by this selective policy implementation.

Uptake of the offer of a 60-hour postpartum stay had much more to do with a woman's own sense of readiness to leave hospital with her infant than with structural factors. A first time mother was more likely to have concerns about leaving hospital and to see the hospital as a "safe haven". She was not obligated to leave hospital by the need to mother other children. Since cost was not a factor at the level of the individual in a system of universal health insurance, these mothers could wait until they believed they were ready to go home.

The question then was asked of whether a lengthening of hospital stay, in the circumstances defined in this study, made any difference in outcomes. Evidence from this study showed no change in the self-evaluated health status of the mother or in her evaluation of her infant's health at 4 weeks post discharge. Similarly, no change was found in infant service utilization of community physicians, emergency rooms, or hospital readmission. Maternal service utilization decreased in relation to the use of community physicians and emergency rooms but readmission increased overall, ranging from 1% to 4% with statistically significant increases at two of the five sites. These findings suggest that, at least in the short term, increasing postpartum LOS increased hospital postpartum care costs without producing substantial health improvement or reduction in costs of service use following discharge.

## Conclusion

The findings of TOMIS and TOMIS II suggest that if practice is to be changed, policy directives, while necessary, may be insufficient incentives. Compelling clinical evidence is required to change practice. Clinicians allocate resources on the basis of their clinical judgement rather than policy guidelines.

Healthy women with healthy babies, when given options, seem to make choices about how long to stay in hospital and choose to stay for the shortest possible period based on their perceptions of their own health status and that of their infant. Flexibility rather than strict rules is needed if both clinical judgement and a woman's preference are to be considered in individual LOS decisions.

Neither health outcomes nor economies in service utilization were found in our study to provide justification for an extended postpartum stay for healthy women and infants. Patient satisfaction may be the most important factor to consider. Women are more satisfied with their hospital experiences when they are offered the option of a stay of up to 60 hours whether or not they stay for this period. Not surprisingly, the ability to exercise some degree of control over one's own care continues to be an important issue in patient satisfaction and probably a factor in a woman's decision about how long to stay in hospital. Therefore, finding optimal lengths of stay for healthy women following an uncomplicated vaginal delivery is appropriately less a matter of policy and more an issue of good clinical judgement on the part of both women and their health care providers.

## Competing interests

The author(s) declare that they have no competing interests.

## Authors' contributions

SW had a major role in designing the study and writing the proposal, co-supervised all aspects of study implementation, participated in data analysis, and was the lead writer of this manuscript.

WS had a major role in designing the study and writing the proposal, co-supervised all aspects of study implementation, participated in data analysis, contributed to the manuscript, and provided editorial comments.

PK contributed to the study design, implementation, analysis and interpretation, as well as the writing of the manuscript.

## Pre-publication history

The pre-publication history for this paper can be accessed here:



## Supplementary Material

Additional file 1 Table – Variables associated with acceptance of a 60-hour length of stay. This table provides the P-value, Unadjusted Odds Ratio, and Confidence Intervals for the variables associated with the acceptance of the offer of a 60-hour postpartum length of stay in hospital. Click here for file
