# Supplementary material for: Longer postpartum hospitalization options – who stays, who leaves, what changes?
Source: BMC Pregnancy Childbirth. 2005 Oct 14;5:13. doi: 10.1186/1471-2393-5-13 (PMC1266374; doi:10.1186/1471-2393-5-13)
Supplement: Additional file 1 — Table – Variables associated with acceptance of a 60-hour length of stay. This table provides the P-value, Unadjusted Odds Ratio, and Confidence Intervals for the variables associated with the acceptance of the offer of a 60-hour postpartum length of stay in hospital. [file 1471-2393-5-13-S1.doc]

**Variables associated with acceptance of a 60-hour length of stay ^a^ (n=379)**

| Variables | Accepted 60 Hr LOS | | P value ^b^ | Unadjusted  Odds Ratio | 95% Confidence Interval |
| --- | --- | --- | --- | --- | --- |
|  | Yes  (%) | No  (%) |  |  |  |
| Information Collected From Mother Prior to Discharge From Hospital | | | | | |
| Age of mother (n=379):  20 to 39 years  <20 or >39 years | 158 (44.4)  14 (60.9) | 198 (39.1)  9 (39.1) | 0.124 | 1.00  1.95 | -  (0.82, 4.62) |
| First live birth (n=379):  No  Yes | 73 (35.6)  99 (56.9) | 132 (64.4)  75 (43.1) | <0.001 | 1.00  2.39 | -  (1.58, 3.61) |
| Has a family physician (n=378):  Yes  No | 166 (45.0)  5 (55.6) | 203 (55.0)  4 (44.4) | 0.737 | 1.00  1.53 | -  (0.40, 5.78) |
| Baby had medical problems since birth (n=379):  No  Yes | 132 (41.0)  40 (70.2) | 190 (59.0)  17 (29.8) | <0.001 | 1.00  3.39 | -  (1.84, 6.23) |
| Number of concerns ^c^ prior to discharge (n=379):  One or fewer  Two or more | 87 (38.2)  85 (56.3) | 141 (61.8)  66 (43.7) | 0.001 | 1.00  2.09 | -  (1.37, 3.17) |
| Mother had medical problems since birth (n=379):  No  Yes | 151 (43.4)  21 (67.7) | 197 (56.6)  10 (32.3) | 0.009 | 1.00  2.74 | -  (1.25, 5.99) |
| Language spoken most often at home (n=379):  Other  English | 21 (38.9)  151 (46.5) | 33 (61.1)  174 (53.5) | 0.301 | 1.00  1.36 | -  (0.76, 2.46) |
| Ethnic or cultural group (n=378):  Canadian  Other | 136 (44.2)  36 (51.4) | 172 (55.8)  34 (48.6) | 0.270 | 1.00  1.34 | -  (0.80, 2.25) |
| Place of birth (n=379):  Canada  Other | 133 (43.5)  39 (53.4) | 173 (56.5)  34 (46.6) | 0.125 | 1.00  1.49 | -  (0.89, 2.49) |
| Marital status (n=378):  Partnered  No Partner | 155 (44.4)  16 (55.2) | 194 (55.6)  13 (44.8) | 0.263 | 1.00  1.54 | -  (0.72, 3.30) |
| Total income before taxes and deductions of all household members (n=351):  $20,000 or more  Less than $20,000 | 135 (44.3)  25 (54.3) | 170 (55.7)  21 (45.7) | 0.200 | 1.00  1.50 | -  (0.80, 2.79) |
| Highest level of education (n=376):  Completed high school or less  Education beyond high school | 34 (42.5)  138 (46.6) | 46 (57.5)  158 (53.4) | 0.510 | 1.00  1.18 | -  (0.72, 1.95) |
| Mother feels that help and support at home will meet both her and baby’s needs (n=379):  Definitely yes  Other | 84 (28.9)  88 (54.0) | 132 (61.1)  75 (46.0) | 0.003 | 1.00  1.84 | -  (1.22, 2.78) |
| Mother feels she and baby are ready to be discharged (n=379):  Definitely yes  Other | 95 (56.9)  77 (36.3) | 135 (63.7)  72 (43.1) | <0.001 | 1.00  2.31 | -  (1.53, 3.50) |
| Information Collected From Mother Four Weeks After Discharge From Hospital | | | | | |
| Number of identified learning needs while in hospital (n=379):  Less than 2  2 or more | 57 (33.1)  115 (55.6) | 115 (66.9)  92 (44.4) | <0.001 | 1.00  2.52 | -  (1.66, 3.84) |
| Mother was offered a home visit (n=332):  No  Yes | 7 (43.8)  153 (48.4) | 9 (56.3)  163 (51.6) | 0.715 | 1.00  1.21 | -  (0.44, 3.32) |
| Mother’s overall rating of hospital labour and delivery services (n=378)  Fair/Poor  Excellent/Good | 9 (36.0)  163 (46.2) | 16 (64.0)  190 (53.8) | 0.323 | 1.00  1.53 | -  (0.66, 3.54) |
| Mother’s overall rating of hospital maternity ward services (n=372):  Fair/Poor  Excellent/Good | 37 (49.3)  134 (45.1) | 38 (50.7)  163 (54.9) | 0.513 | 1.00  1.18 | -  (0.71, 1.97) |
| Mother’s rating of her own health since having the baby (379):  Excellent/Very good  Good/Fair/Poor | 92 (40.4)  80 (53.0) | 136 (59.6)  71 (47.0) | 0.016 | 1.00  1.67 | -  (1.10, 2.52) |
| Confidant Support Score ^c^ (n=377):  20 to 25  5 to 19 | 124 (43.1)  48 (53.9) | 164 (56.9)  41 (46.1) | 0.072 | 1.00  1.55 | -  (0.96, 2.50) |
| Affective Support Score ^d^ (n=377):  12 to 15  3 to 11 | 151 (44.0)  21 (61.8) | 192 (56.0)  13 (38.2) | 0.048 | 1.00  2.05 | -  (1.00, 4.24) |
| Edinburgh Postnatal Depression Scale Score (n=379):  <12  12 or more | 154 (44.3)  18 (58.1) | 194 (55.7)  13 (41.9) | 0.139 | 1.00  1.74 | -  (0.83, 3.67) |
| Mother’s rating of infant’s health since discharge (n=378):  Excellent/Very good  Good/Fair/Poor | 143 (44.4)  29 (51.8) | 179 (55.6)  27 (48.2) | 0.306 | 1.00  1.34 | -  (0.76, 2.37) |
| Mother can tell when baby is sick (n=378):  Yes, most of the time  Sometime/No/ DK | 126 (42.1)  46 (58.2) | 173 (57.9)  33 (41.8) | 0.011 | 1.00  1.91 | -  (1.16, 3.16) |
| Any form of breast feeding initiated (n=378):  No  Yes | 19 (35.2)  153 (47.2) | 35 (64.8)  171 (52.8) | 0.100 | 1.00  1.65 | -  (0.91, 3.00) |

^a^ The exact question asked was “Did you use this option? Yes/No”. It followed the question “Were you offered the option of a 60-hour stay in hospital after your delivery? Yes/No”.

^b^ Chi-square test

^c^ Concerns included: breast-feeding; bottle-feeding; infant care and behaviour; signs of illness in infant; physical changes and care of yourself; sexual changes and intercourse; emotional changes in yourself.

^d^ From “The Duke-UNC Functional Social Support Questionnaire”
